# Physical Exercise Mitigates Salivary Gland and Saliva Damages in Rats Exposed to Binge-like Ethanol Pattern

**DOI:** 10.3390/antiox12051038

**Published:** 2023-05-03

**Authors:** Beatriz Rodrigues Risuenho Peinado, Walessa Alana Bragança Aragão, Cristian dos Santos Pereira, Diane Cleydes Baia da Silva, Railson de Oliveira Ferreira, Antônio Hernandes Chaves-Neto, Luanna Melo Pereira Fernandes, Rafael Rodrigues Lima

**Affiliations:** 1 Laboratory of Functional and Structural Biology, Institute of Biological Sciences, Federal University of Pará, Belém 66075110, Brazil; 2 Department of Basic Sciences, School of Dentistry, São Paulo State University (UNESP), Araçatuba 16066840, Brazil; 3 Department of Morphology and Physiological Sciences, Center of Sciences Biological and Health, State University of Pará, Belém 66087662, Brazil; luannafe@hotmail.com

**Keywords:** alcohol, binge-drinking, salivary gland, moderate physical training, oxidative biochemistry

## Abstract

Heavy episodic ethanol (EtOH) consumption is a typical pattern, especially among younger people. The therapeutic effect of exercise on EtOH damage has not yet been fully elucidated. Therefore, this study aims to investigate whether moderate exercise can reduce the damage generated by ethanol consumption in salivary glands and saliva. Thus, 32 male Wistar rats were divided into four groups: control (sedentary animals treated with water); training (trained animals treated with EtOH); EtOH (sedentary animals treated with EtOH); and EtOH + training (trained animals treated with ethanol). EtOH was administered to the animals at a dose of 3 g/kg/day at a concentration of 20% *w/v* for three consecutive days per week via intragastric gavage. The training was performed on a treadmill for five successive days. At the end of the 4-week experimental protocol, the animals were euthanized, and salivary glands and saliva were collected for oxidative biochemistry analysis. Our results showed that EtOH consumption generated changes in the oxidative biochemistry of the salivary glands and saliva. Thus, it was possible to conclude that moderate physical exercise can significantly recover antioxidant activity, reducing the damage generated by EtOH.

## 1. Introduction

Ethanol (EtOH) is the most consumed substance worldwide in the form of alcoholic beverages. Its consumption has been favored by numerous cultures and has motivated celebrations for centuries [1]. The drug’s psychoactive properties promote its harmful use, such as alcohol abuse leading to alcohol-use disorder, which is associated with deleterious effects on personal health [2,3]. These detrimental effects of alcohol misuse have significant social and economic consequences [1].

**Figure 1:**
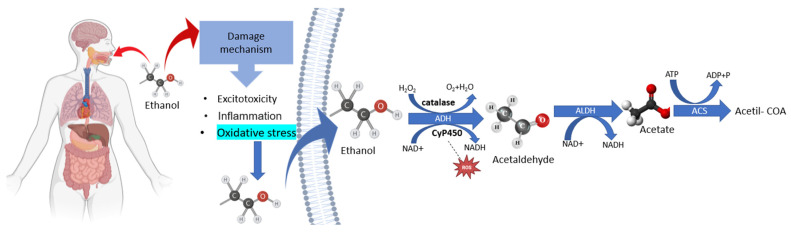
Representative schematic of the toxic damaging mechanisms of ethanol (cytotoxicity, inflammation, and oxidative stress) and its hepatic metabolization in the human body. The enzyme alcohol dehydrogenase (ADH) oxidizes ethanol to acetaldehyde. This, in turn, undergoes the action of aldehyde dehydrogenase (ALDH), generating the acetate molecule that participates in several metabolic cycles.

It is known that the harmful effects of alcohol frequently vary according to dose and consumption period. Among patterns of alcohol intake, binge drinking is defined as the ingestion of four (women) to five drinks (men) in a 2 h time frame in the past 30 days, which brings about a blood alcohol concentration of 0.08 mg per deciliter or higher [4,5]. Heavy drinking is also an alcohol use disorder defined by five or more times the gender-specific binge threshold [6]. Those patterns are considered a health issue worldwide due to their association with injuries, violence, and chronic diseases. In general, despite consumption patterns, ethanol is well absorbed orally, freely passes through membranes, and is well distributed throughout the body without binding to any plasma proteins [7]. EtOH undergoes hepatic metabolization mainly by an oxidative pathway which is oxidized to acetaldehyde primarily by alcohol dehydrogenase (ADH). However, this oxidation may occur by means of the hepatic microsomal ethanol oxidation system (MEOS)—CYP450 2E1—and catalase (Figure 1) [8]. The second step in the oxidative pathway involves the action of aldehyde dehydrogenase (ALDH) converting the acetaldehyde to acetate, which is harmless [3,9].

If heavier EtOH consumption occurs, increases in the expression and activity of MEOS generate the production of acetaldehyde through the formation of reactive oxygen species (ROS), such as radical superoxide anion (O_2_^−^) and hydrogen peroxide (H_2_O_2_) [9]. Acetaldehyde is the most toxic metabolite produced from alcohol metabolism; it interacts with DNA, causing mutations and chromosomal damage, positively regulating CYP2E1 expression, and increasing oxidative stress [3,9]. Then, this system produces additional reactive oxygen radicals, possibly damage mitochondria and, thus, generating cytotoxicity, inflammation, and cell death [5,8,10].

Oxidative stress is characterized by an imbalance of the redox system, elevated levels of free radicals, and an impaired antioxidant system [8]. Studies have revealed that a deficiency of the essential vitamins for the production of the potent antioxidant glutathione, mainly vitamins B1 (thiamine), C, B9 (folate), and B12, are commonly observed in cases of alcohol abuse [3,11]. Therefore, oxidative stress is linked to the etiology of many diseases and plays a vital role in the damage mechanism of alcohol disorders [8]. Therefore, high blood alcohol concentration quickly reaches the body tissues and disrupts their functioning, especially the liver, central nervous system, bone, and cellular metabolisms [7,10]. Thus, alcohol use disorders are associated with the risk of alcoholic liver disease, cardiovascular diseases, pancreatitis, cancers, malnutrition problems, brain damage, and fetal alcohol spectrum disorders [3,8,9]. Interestingly, salivary glands are susceptible to biochemical, morphological, and functional damage through different forms of alcohol exposure, both in chronic and binge-like models [12,13,14].

Physical activity is defined as any skeletal muscle-driven movement that requires more energy expenditure than usual. For example, it includes tasks related to everyday life, recreation, employment, sports, and active transportation [15,16]. In contrast, methodological factors of physical training, such as volume, intensity, frequency, and type of exercise, define exercise as a planned and structured activity with the primary goal of improving the body’s capabilities. Both types of activity have a preventive impact against several diseases and play a role in redox balance [17].

In addition, exercise is an efficient strategy to minimize alcohol-derived central nervous system impairments [18,19]. A recent study found a relationship between exercise and salivary oxidative stress, especially at moderate intensity (see the systematic review of Alves et al., 2022 [20]). However, the interaction between EtOH consumption and the effects of moderate-intensity physical exercise on the salivary glands and saliva remains unclear in the literature. Thus, this study aimed to evaluate whether physical exercise can prevent the damage caused by binge-like EtOH patterns in Wistar rats.

## 2. Materials and Methods

### 2.1. Animals and Experimental Groups

This experiment was conducted with the approval of the Ethics Committee on Experimental Animals of the Federal University of Pará, number 1423181219. A total of 32 male albino rats (*Rattus norvegicus*) species, Wistar strain, 90 days old, were used. The animals were housed in plastic collective cages (40 cm × 30 cm × 13 cm), with 4 animals per cage at a temperature of 25 °C on a 12 h dark/light cycle. For four weeks, the animals were fed a balanced diet and had access to water *ad libitum*.

The animals were divided into four groups, with eight animals each, as follows: control group (sedentary animals that received distilled water); training group (trained animals that received distilled water); EtOH group (sedentary animals treated with ethanol); and EtOH + training group (trained animals treated with ethanol).

An evaluation of their trainability was performed on a treadmill to help distribute the animals among the groups, and a scale of 1 to 5 was used [21]. In this way, each animal was observed during a 5 min run at a speed of 15 m/min and, according to its performance, received one of the five classifications below:

1. The animal refuses to run;
2. Running without constancy (runs and stops or runs in the wrong direction);
3. Regular race;
4. Good run (occasionally runs at the back of the treadmill);
5. Excellent running (runs permanently at the front of the treadmill).

Animals that received a rating equal to or greater than three were elected for the physical training group. In contrast, animals with a rating of 1 to 2 were selected for the sedentary animal groups. In this way, it was possible to reduce the bias, minimizing possible differences related to the level of stress that each animal suffered [22].

### 2.2. Binge-like EtOH Treatment

**Figure 2:**
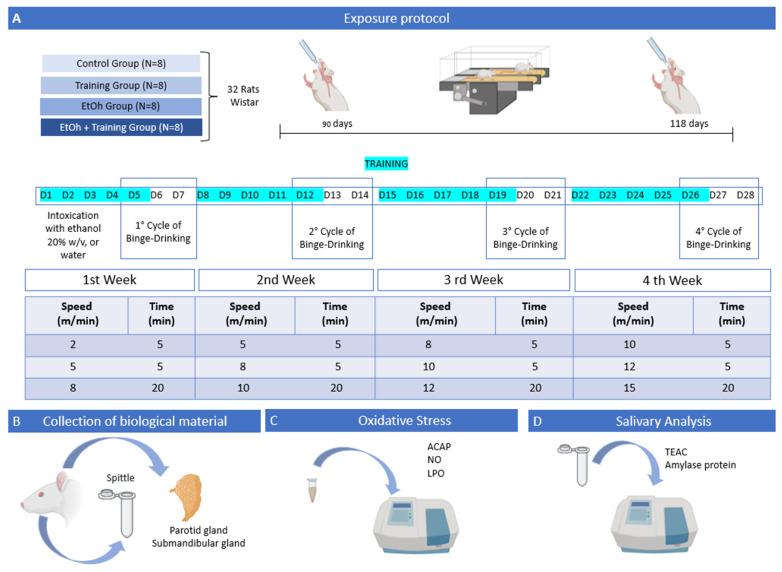
Summary of the methodology used in the experiment, referring to the period of training and intoxication, removal of salivary glands, and analyses performed. Thirty-two male Wistar rats were divided into four groups of eight animals. Two groups were submitted to thirty minutes of forced running on five consecutive days a week for four weeks. All groups were administered distilled water or EtOH at the end of each week (**A**). After 28 days of the experiment, the animals were anesthetized for the collection of saliva and salivary glands (**B**). The glandular tissue was used to determine oxidative stress through analysis of antioxidant capacity against peroxyl radicals (ACAP), nitrite concentration (NO), and lipid peroxidation assay (LPO) (**C**), and saliva was analyzed by assessing Trolox-equivalent antioxidant capacity level (TEAC) and amylase activity (**D**).

The animals in the EtOH and EtOH + training groups were intoxicated with ethanol at a dose of 3 g/kg/day (20% *w/v*) orally (gavage) through an orogastric cannula (Insight, Brazil) on three consecutive days per week for four weeks [13,23], while the control group and the sedentary + water group received distilled water by the same route to simulate the same level of stress naturally generated by the gavage process. The animals were weighed weekly to adjust the dose. On the fifth day of each week, EtOH or distilled water was administered eight hours after the last training day. Thus, the animals underwent twelve binge sessions throughout the entire experiment. The experimental design is summarized in Figure 2.

### 2.3. Aerobic Physical Training Protocol

The training protocol was performed according to the model of Arida et al. (2011) [24], modified and validated by Pamplona-Santos et al. (2019) [19] and Lamarão-Vieira et al. (2019) [18]. It was characterized as moderate-intensity aerobic exercise, in which the animals were forced to walk on a treadmill adapted for rodents with no inclination degree (Insight LTDA). Each animal was separated from the others by an acrylic wall. The exercise lasted 30 min daily for 5 consecutive days of the 4-week experiment. The treadmill’s speed was modified weekly, following the scale shown in Figure 2.

### 2.4. Collection of Saliva and Salivary Glands

At the end of 4 weeks of administration with EtOH associated with physical exercise on a treadmill, the animals were anesthetized with a combined solution of ketamine hydrochloride (90 mg/kg) and xylazine (9 mg/kg). Pilocarpine (1 mg/kg) was injected intraperitoneally to collect saliva in plastic microtubes over ice for 10 min, with immediate storage of the samples at −80 °C. Then, the animals were euthanized for resection of the parotid and submandibular glands, which were washed in saline solution and subjected to freezing with liquid nitrogen, then stored at −80 °C until the time of biochemical analysis. Subsequently, to prepare the homogenates of the salivary gland samples, sonic disaggregation was performed in 20 mmol/L Tris-HCl, pH 7.4 at 4 °C (approximate concentration of 1 g/mL). The supernatants were collected, and aliquots were separated for each biochemical analysis.

### 2.5. Salivary Gland Oxidative Biochemistry Analysis

#### 2.5.1. Analysis of Antioxidant Capacity against Peroxyl Radicals (Antioxidant Capacity against Peroxyl Radicals—ACAP)

For this analysis, the samples were centrifuged at 14,000 rpm (Eppendorf Centrifuge 5910 Ri) to obtain the homogenate. In this method, quantification of ROS generated by equally concentrated samples (2.5 g protein/L) after exposure to a peroxyl radical generator was used to assess ACAP [25]. Thermal cracking of 2,2′-azobis 2-methylpropionamidine dihydrochloride at 35 °C resulted in the production of peroxyl radicals (ABAP; 4 mmol/L; Sigma-Aldrich, St. Louis, MI, USA). The 2′,7′ dichlorofluorescein diacetate (H_2_DCF-DA, InvitrogenTM, Whaltan, MA, USA) was used for ROS detection at a final concentration of 40 nmol/L. Every 5 minutes for 30 minutes, measurements were made in a fluorescence microplate reader (Victor X3, Perkin Elmer, Waltham, MA, USA). The relative difference between the area of ROS with and without ABAP was considered as a measure of antioxidant capacity. From this, the results were converted to the inverse of the relative area and expressed as percentages of the control.

#### 2.5.2. Lipid Peroxidation Assay

Malondialdehyde (MDA) levels were used as an indication to calculate LPO levels in the salivary glands [26]. Homogenized samples were centrifuged at 5600 rpm (Eppendorf Centrifuge 5910 Ri) for 10 min at 4 °C. The next step involved the incubation of sample supernatants and standard MDA solutions with a 10.3 mmol/L N-methyl-2-phenylindole solution diluted in methanol (1:3) and methanesulfonic acid at 45 °C for 40 min. Then, a spectrophotometric reading was performed (λ = 570 nm). Results are plotted as percent of the control, and are represented in nanomolars per microgram of protein (nmol/µg). The Bradford method was used to quantify proteins [27].

#### 2.5.3. Analysis of Nitrite Concentration

For this determination, the lysate was thawed and centrifuged at 14,000 rpm (Eppendorf Centrifuge 5910 Ri) for 10 min at 4 °C. The supernatant was separated into aliquots to determine nitrite and protein concentrations. The concentration of nitrites was determined based on their reaction with the Griess reagent (Naphthyl-ethylene-diamine 0.1% and sulfanilamide 1% in phosphoric acid 5%-1:1). This reaction formed azoic compounds, which yield a characteristic bluish color. The intensity of the blue color was proportional to the chromogen concentration, and was verified by spectrophotometric reading [28]. Afterwards, 50 microliters of supernatant or standard nitrite solution were added to 100 μL of Griess reagent and incubated for 20 min at room temperature. Afterward, the reading was performed (λ = 550 ηm), and the results were expressed as micromoles of nitrites for each microgram of proteins (µmol/µg).

### 2.6. Saliva Biochemical Analysis

#### 2.6.1. Trolox-Equivalent Antioxidant Capacity (TEAC) Level

This analysis was performed according to the methodology outlined by Re et al. 1999 [29], with modifications. It was based on the ability of substances to eliminate the ABTS^•+^ radical cation, a blue-green chromophore that reduces its intensity in the presence of antioxidants, resulting in the formation of ABTS, which can be identified by its characteristic colorlessness. The ABTS^•+^ solution (2.45 mmol/L) was prepared through the reaction between ABTS (7 mmol/L) and potassium persulfate (140 mmol/L; K_2_O_8_S_2_). Initially, the reading (T0) of the ABTS^•+^ solution was performed. Then, 30 μL of sample or standard was added to this solution. After 5 min, a second reading was performed (T5). The reaction was measured by a spectrophotometer at 734 nm. The results were expressed in mmol per liter (mmol/L).

#### 2.6.2. Amylase Activity

Saliva samples were weighed, frozen in liquid nitrogen, and stored at −80 °C. To determine the amylase activity, the K003 BIOCLIN^®^ colorimetric amylase test kit (QUIBASA, Química Básica Ltd.a., Belo Horizonte, Minas Gerais, Brazil) was used according to the modified Caraway method [30]. Absorbances were measured in a spectrophotometer at 660 nm. Amylase activity results were expressed as U/µg of salivary protein.

### 2.7. Statistical Analysis

After data collection, the distribution was tested using the Shapiro–Wilk method to verify normality. Statistical comparisons between groups were performed using one-way ANOVA and Tukey’s post hoc test. Values of *p* < 0.05 were considered statistically significant. All analyses were performed using GraphPad Prism 7.0 software (San Diego, CA, USA).

## 3. Results

### 3.1. Physical Training Was Able to Attenuate the Oxidative Stress Generated by the EtOH Consumption in the Salivary Glands in a Binge-Drinking Model in Rats

**Figure 3:**
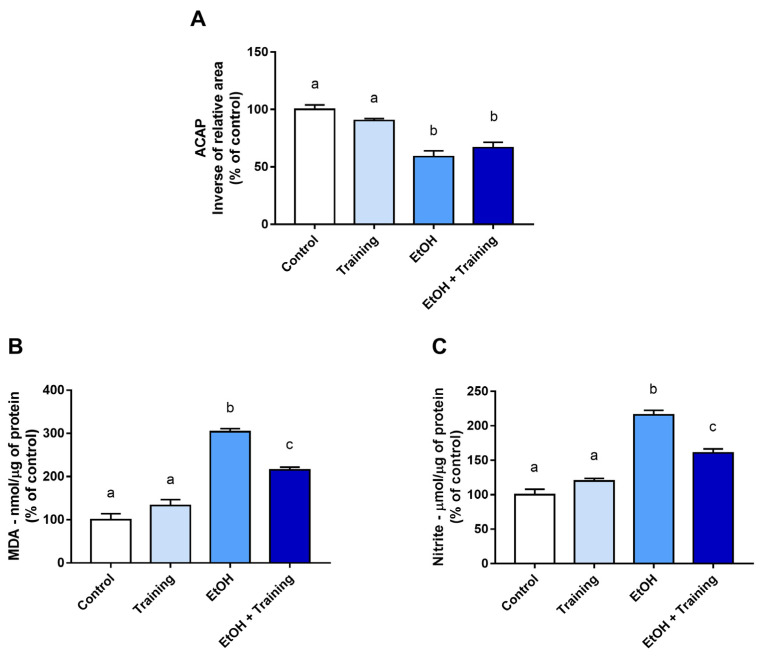
Parameters of the oxidative biochemistry of the parotid glands after intense and episodic ingestion of ethanol (binge drinking pattern) in sedentary and trained Wistar rats for 90 days. In (**A**), antioxidant capacity against peroxyl-ACAP radicals. (**B**) Lipid peroxidation concentration (LPO); (**C**) nitrite concentration. Results are expressed as mean ± standard error of the mean and converted to percentages of the control. One-way ANOVA and Tukey’s post hoc test were used. Different superscript letters between groups indicate statistical differences (*p* < 0.05).

In the parotid gland, EtOH reduced the concentration of antioxidant capacity compared to the groups that did not ingest EtOH (*p* < 0.0001), and physical training was not able to minimize the damage to this parameter (Figure 3A).

On the other hand, when evaluating the LPO levels in the parotid gland, a significant increase was observed in the groups that received the binge-like EtOH treatment compared to those treated with distilled water only (*p* < 0.0001). Moreover, the physical training alone did not change this parameter compared to the control group (control group means: 100%; training group means: 132.7%; *p* = 0.1847). However, physical training reduced LPO levels compared to the sedentary group that received EtOH (EtOH group mean: 303.7%; EtOH + training group mean: 215.5%; *p* < 0.0001; Figure 3B).

A significant increase in nitrite levels in the parotid gland was observed in the groups that received treatment with EtOH (*p* < 0.0001). However, when analyzing the isolated effect of physical training on this parameter compared to the control group, it was not observed significant changes (control group mean: 100%; training group mean: 119.9%; *p* = 0.1376). On the other hand, physical training was able to reduce nitrite levels compared to the sedentary group that received EtOH (EtOH group mean: 216%; EtOH + training group mean: 160.8%; *p* < 0.0001; Figure 3C).

**Figure 4:**
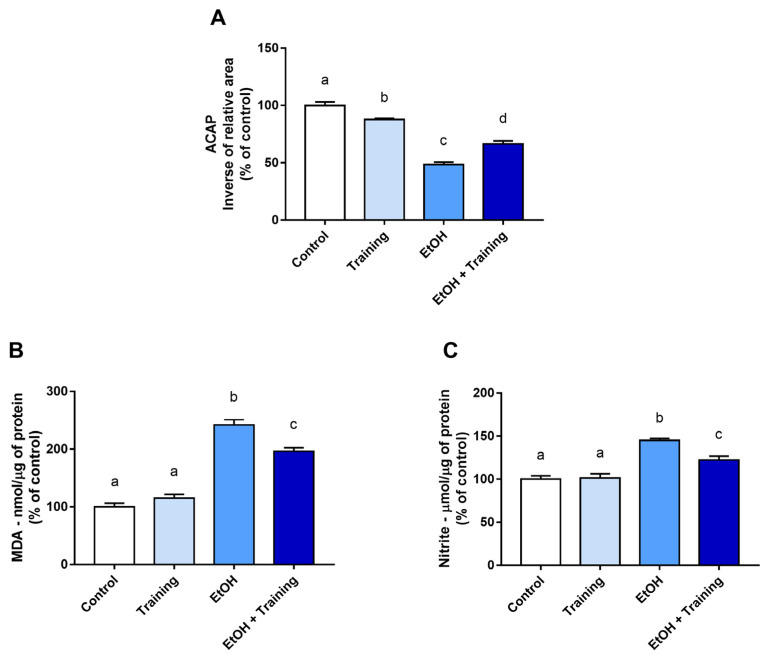
Parameters of the oxidative biochemistry of the submandibular glands after intense and episodic ingestion of ethanol (binge drinking pattern) in sedentary and trained Wistar rats for 90 days. (**A**) Antioxidant capacity against peroxyl -ACAP radicals; (**B**) nitrite concentration; (**C**) lipid peroxidation concentration (LPO). Results are expressed as mean ± standard error of the mean and converted to percentages of the control. One-way ANOVA and Tukey’s post hoc test were used. Different superscript letters between groups indicate statistical differences (*p* < 0.05).

Meanwhile, in the submandibular glands, moderate-intensity aerobic physical training decreased oxidative stress by modulating all evaluated parameters. When assessing the ACAP parameter, it was observed that the consumption of EtOH promoted a reduction in the antioxidant capacity compared to the groups that received only distilled water (*p* < 0.0001). Regarding the effect of physical training, it was verified that the protocol application still failed to recover the control levels (control group means: 100%; training group means: 87.63%; *p* = 0.0060). However, physical training generated a recovery effect on ACAP levels compared to the sedentary group that received the binge-like EtOH treatment (EtOH group mean: 48.41%; EtOH + training group mean: 66.39%; *p* < 0.0001; Figure 4A).

Regarding the LPO parameter, EtOH promoted an increase in MDA levels compared to the groups that received only distilled water (*p* < 0.0001), and the effect of physical training alone did not cause changes in this parameter (control group mean: 100%; training group mean: 115%; *p* = 0.4552). However, when evaluating the LPO levels in the groups subjected to binge-like EtOH treatment, a significant reduction was observed in the group that received the aerobic physical training protocol (EtOH group mean: 242%; EtOH + training group mean: 196.4%; *p* = 0.0007; Figure 4B).

Similarly, nitrite levels in the submandibular gland were elevated in the groups that received the binge-like EtOH treatment (*p* < 0.0001), and physical training was able to reduce nitrite levels compared to the sedentary group that consumed EtOH (EtOH group mean: 145%; EtOH + training group mean: 122.3%; *p* = 0.0032; Figure 4C).

### 3.2. The Intense and Episodic EtOH Consumption Promoted Alterations in the Salivary Oxidative Biochemistry of the Animals, but Aerobic Physical Training Could Not Minimize the Damage

**Figure 5:**
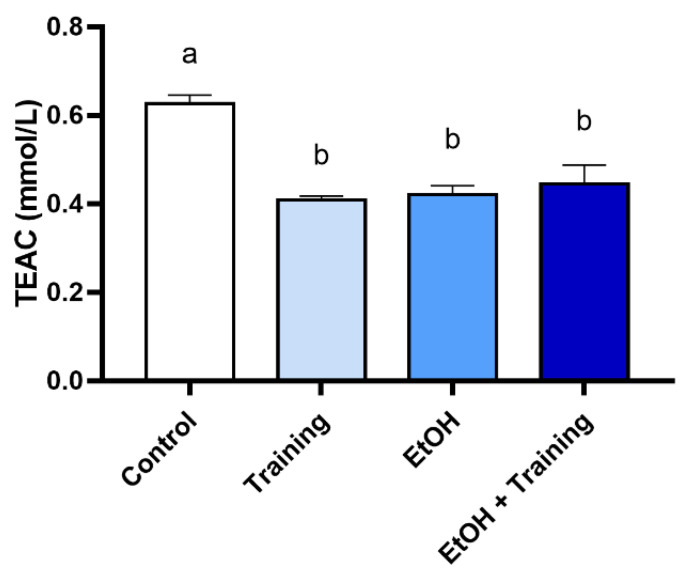
Salivary Trolox-equivalent antioxidant capacity (TEAC) after intense and episodic ethanol ingestion (binge drinking pattern) in sedentary and trained Wistar rats for 90 days. Trolox-equivalent antioxidant capacity (TEAC) levels are shown. Results are expressed as mean ± standard error of the mean. One-way ANOVA and Tukey’s post hoc test were used. Different superscript letters between groups indicate statistical differences (*p* < 0.05).

**Figure 6:**
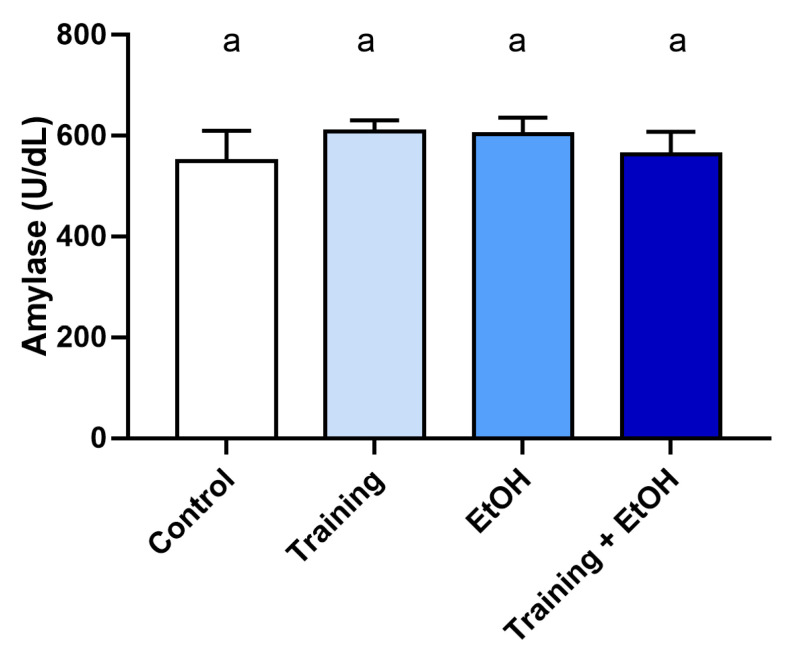
Salivary amylase activity after intense and episodic ethanol ingestion (binge drinking pattern) in sedentary and trained Wistar rats for 90 days. Results are expressed as mean ± standard error of the mean. One-way ANOVA and Tukey’s post hoc test were used. Different superscript letters between groups indicate statistical differences (*p* < 0.05).

The results showed that TEAC levels in the EtOH group were lower compared to the control group (*p* < 0.0001), but physical training was not able to minimize the modulation generated (*p* > 0.05; Figure 5). On the other hand, exposure to EtOH in the binge drinking model did not cause alterations in the amylase activity in the saliva of rats (*p* > 0.05; Figure 6).

All significantly relevant values from the biochemical and saliva analyses are contained in Supplementary Tables S1 and S2.

## 4. Discussion

This study demonstrated that EtOH induced alterations in the oxidative biochemistry of the parotid and submandibular glands, suggesting an association with oxidative stress, as indicated by the decreased antioxidant capacity (ACAP) and increased pro-oxidant parameters (LPO and NO). On the other hand, physical exercise was able to promote a reduction in pro-oxidant parameters in salivary glands and the recovery of antioxidant levels in the submandibular gland. Evidently, EtOH modified salivary antioxidant capacity (TEAC); however, exercise did not have the same beneficial effect on salivary glands, as antioxidant levels did not recover. In addition, amylase activity did not change significantly after EtOH consumption (supplementary Table S2).

In this context, it is necessary to approach the morphology and physiology of the salivary glands, as they are composed of different cell types, including endothelial, mesenchymal, and epithelial neuronal cells [31]. The major salivary glands can be defined as agglomerations of acini in arborized form, which act as secretory units; a complex system of ducts, which contribute to the salivary formation and transport; and myoepithelial cells, which are involved in salivary secretion [32,33]. Classified as exocrine, these glands are present in the oral cavity as three pairs of major glands (parotid, submandibular, and sublingual) and several minor glands which are distributed in some oral mucosa regions, such as the cheeks and lips [34,35,36].

In rats, the parotid glands are composed only of serous cells, while the submandibular glands are composed of both serous and mucous acinar cells, although the presence of serous acini is predominant. Parotid serous cells secrete a large number of proteins such as amylase, resulting in the production of more watery saliva. On the other hand, the mixed submandibular acini secrete more viscous saliva due to a large number of glycoproteins [32,34,36]. Therefore, due to these different biochemical and morphological characteristics, each gland plays an individual role in the production and composition of saliva [37]. In this way, saliva performs numerous functions which are essential for maintaining oral and systemic health, such as protection and cleaning of the teeth and mucosa, antimicrobial action, feeding and digestion facilitation, and maintaining pH. Therefore, quantitative and qualitative losses in this fluid can cause discomfort, such as taste changes and difficulty in chewing and swallowing, as well as favoring the increase in carious lesions and pathological conditions such as candidiasis and mucositis [38,39]. In addition, salivary biomarkers can contribute to diagnosing oral and systemic disorders [40]. Therefore, it is relevant to understand the possible etiologies of the alterations that occur in the salivary glands and, in particular, how intense and episodic ethanol consumption can modulate the behavior of this tissue.

Our findings showed a significant increase in oxidative stress parameters and a similarly reduced antioxidant capacity induced by intense and episodic consumption of ethanol (3 g/kg/day) in both glands. This alteration could be observed through reduced levels of ACAP and higher levels of LPO and nitrite in the groups exposed only to ethanol (supplementary Table S1). These results are compatible with other studies which observed oxidative damage in female adolescent rats after ethanol consumption. This effect occurred due to increased levels of lipid peroxidation markers, determined through MDA levels in the tissues studied, in the parotid glands after 1 and 4 weeks of binge drinking, in the submandibular glands after only one week of binge drinking [13], and in the motor cortex after four weeks of binge drinking. This was in addition to a reduction in the activity of the catalase (CAT) and superoxide dismutase (SOD) antioxidant enzymes, unbalancing the protection system against damage caused by oxidative stress [23].

Another study evaluated the impact of ethanol consumption on the salivary glands through saliva analysis, and also observed alterations in the functional biochemical system in response to the toxic effect of ethanol through assessment of the enzymatic activity of total acid phosphatase (TAP) and tartrate-resistant acid phosphatase (TRAP). The researchers noticed a progressive reduction in both activities in the submandibular glands of rats exposed to chronic consumption of cachaça for 75–105 days, in addition to reduced mucin levels in the exposed group [12]. The metabolization of ethanol produces acetaldehyde, which is linked to greater production of ROS. This contributes to cellular oxidation and, consequently, the damage caused by this process [41]. These analyses demonstrated that these toxic effects of ethanol consumption affect the biochemical system in different and time-dependent ways [13,23,41].

In this context, physical exercise minimized the oxidative imbalance in the parotid and submandibular glands, where recovery of the antioxidant capacity expressed by ACAP was observed. This analysis method detects ROS by fluorescence, so a peroxyl radical generator is added to the samples, which should intercept these ROS as a compensatory response to biochemical imbalance [25]. This damage reduction may be associated with increased blood flow in the salivary glands. For instance, according to a study conducted in an experimental model with rats [42], voluntary physical training on the wheel induced increased expression of angiogenesis markers VEGF-1 and CD-31 in the submandibular gland. Thus, a greater local blood flow would increase the concentration of endogenous and exogenous antioxidants in the gland, which would consequently decrease the concentration of ROS. However, the damage caused by EtOH may have altered the homeostatic dynamics of the salivary glands, as was also observed in the analysis of LPO and nitrite levels, both pro-oxidant parameters. The LPO levels were increased with the consumption of EtOH, and this parameter indicates the occurrence of oxidation of lipid membranes in the cells, along with the release of metabolites such as malondialdehyde, which is considered a biomarker of plasma damage [26]. Besides this parameter, the analysis of nitrite is an indicator of redox changes, because it is a method that quantifies a metabolite of nitric oxide [43] which showed increased levels after EtOH consumption. However, physical exercise contributed to reductions in both pro-oxidant parameters. Corroborating our findings, another study showed that aerobic exercise in aged rats decreased ROS generation and oxidative DNA damage markers in the salivary gland while significantly recovering the salivary flow rate and total protein compared to young rats [44].

It is evidenced in the literature that in this experimental model, in which ethanol was administered and absorbed directly into the digestive tract, ethanol consumption causes alterations in the peripheral blood. It also generates repercussions in the hippocampus of rats, such as reduced levels of total antioxidant capacity (TEAC) and glutathione (GSH) and increased concentrations of MDA and nitrite in hippocampal tissue, in addition to reduced levels of GSH in plasma. These are the parameters that were restored by physical training [19].

On the other hand, the literature points out that physical exercise naturally promotes increases in oxidative stress through several pathways. One of them is due to the increase in intramuscular calcium concentration during physical activity, which promotes the formation of superoxide and hydroxyl radicals, the latter being the most reactive of the intermediary radicals. Another example is leukocyte activation in response to exercise-induced muscle damage, since neutrophils can reduce molecular oxygen into superoxide radicals [45,46]. However, studies also show that while exercise promotes an increase in oxidative stress, it also increases the expression of antioxidant defense regulators, such as heme oxygenase-1 enzyme and the peroxisome proliferator-activated receptor γ coactivator 1α [47]. In addition, exercise promotes compensatory adaptation, increases systemic antioxidant capacity, and consequently improves the ability to reduce free radical reactivity [48].

Thus, the physical exercise protocol in this study was configured as a moderate-intensity model of aerobic resistance, a strategy for maintaining quality of life and preventing chronic diseases [18,19]. Its effective role in minimizing oxidative damage caused by EtOH has been evidenced in previous studies which observed reductions in oxidative stress in different regions of the central nervous system, such as the hippocampus [19,49] and cerebellum [18]. Furthermore, the systemic changes caused by EtOH were expressed through the analysis of redox parameters in blood, where exercise increased antioxidant activity [19,50].

Through the capacity of physical exercise to act systemically, the possible mechanisms involved in this process include the regulation of the redox and anti-inflammatory systems [51]. Some studies have reported a decrease in the production of ROS and pro-inflammatory cytokines (TNF-α and IL-1β). At the same time, mitogen-activated protein kinase (MAPK) activation may occur, which promotes the induction of the NF-κB pathway and results in increased antioxidant defenses [52]. Another study showed that exercise increased antioxidant activity expressed by superoxide dismutase (SOD), catalase (CAT), and glutathione peroxidase (GPx) [53]. Similarly, such a modulating effect can be identified by observing reductions in lipid peroxidation in the liver mitochondria of trained rats [54,55]. This increase in antioxidant defense in response to physical exercise was also observed in saliva analysis, and was determined by an increase in total antioxidant activity and a decrease in the concentration of lipid hydroperoxides (oxidative stress index) immediately after physical exercise [56].

In the salivary glands, the effects of EtOH were significant to the extent that they altered the homeostasis of the redox system. In addition to this are the alterations found in the saliva, which were expressed by reductions in antioxidant capacity (TEAC) in the groups that ingested EtOH. However, regarding the role of physical exercise, it can be observed that it did not promote a compensatory response of increased antioxidant capacity in the saliva, contrary to what was observed in the salivary glands. Such a finding may be related to the fact that the oral cavity is more exposed to acetaldehyde, the concentration of which is associated with EtOH-induced oxidative stress [57,58]. In this context, salivary acetaldehyde concentrations after ethanol ingestion reflect its production by alcohol dehydrogenases in the epithelium of the oral mucosa, especially at higher concentrations, and EtOH oxidation by the oral microbiota [59]. Therefore, overexposure to acetaldehyde in the oral cavity of trained animals may be responsible for mitigating the beneficial effects of training on salivary TEAC.

Furthermore, amylase activity, a marker of salivary stress through sympathetic activation, was evaluated [60]. It can be observed that there was no significant change in enzyme activity after moderate exercise, which corroborates the findings of a study that also applied a moderate exercise model at 75% VO_2_ max and did not find changes in amylase [61]. Another study also showed similar results in a model of voluntary wheel running with mice, in which no significant differences in amylase activity nor in the flow rate between groups were observed [62]. This outcome may have resulted from resting after exercise, during which the amylase concentration returns to baseline levels [63].

Thus, despite the limitations of our study, essential data compatible with the literature were found regarding how moderate physical exercise can reduce the damage generated by binge drinking. Therefore, more research is required to determine whether this behavior can affect other salivary functions, whether other training protocols can produce similar or better results, and how they can be used as therapeutic measures for this problem, given the local and systemic harm that heavy ethanol consumption can cause.

## 5. Conclusions

The EtOH binge-drinking consumption pattern promoted changes in the redox system of rats’ parotid and submandibular glands with reduced ACAP and increased levels of LPO and nitrite. Furthermore, it was possible to identify a reduction in the antioxidant capacity in saliva without altering the amylase activity. In contrast, moderate-intensity aerobic physical exercise proved to be a therapeutic tool with beneficial effects. This is because it promoted the recovery of antioxidant capacity and reduced pro-oxidant parameters in both glands, favoring improvement in redox homeostasis animals subjected to EtOH binge-like exposure.

## Data Availability

All data are available within the article.

## Supplementary Materials

The following supporting information can be downloaded at: https://www.mdpi.com/article/10.3390/antiox12051038/s1, Table S1: “Biochemical analysis of the nitrite levels (NO), lipid peroxidation levels (LPO), and antioxidant capacity against peroxyl (ACAP) of parotid and submandibular glands of rats.” Table S2: “Amylase analysis and trolox-equivalent antioxidant capacity (TEAC) of saliva of rats”.
