# Supplementary material for: Physical Exercise Mitigates Salivary Gland and Saliva Damages in Rats Exposed to Binge-like Ethanol Pattern
Source: Antioxidants (Basel). 2023 May 3;12(5):1038. doi: 10.3390/antiox12051038 (PMC10215489; doi:10.3390/antiox12051038)
Supplement: Supplementary file 1 [file antioxidants-12-01038-s001.zip › antioxidants-2280940-supplementary.pdf]

Supplementary Table S1: Biochemical analysis of the nitrite levels (NO), lipid peroxidation levels (LPO), and antioxidant capacity against peroxyl (ACAP) of parotid and submandibular glands of rats. Results are presented as mean ± standard error of mean.

**PAROTID**			
	**NO**	**LPO**	**ACAP**
**Control**	100 ± 8.083%	100 ± 14.07%	100 ± 3.889%
**Training**	119.9 ± 3.679%	132.7 ± 13.94%	90.34 ± 1.777%
**EtOH**	216 ± 6.627%	303.7 ± 7.55%	58.88 ± 5.197%
**Training + EtOH**	160.8± 5.778%	215.5 ± 6.371%	66.69 ± 4.764%

**SUBMANDIBULAR**			
	**NO**	**LPO**	**ACAP**
**Control**	100 ± 3.982%	100 ± 6.431%	100 ± 3.051%
**Training**	101.3 ± 4.957%	115 ± 6.614%	87.63 ± 1.163%
**EtOH**	145 ± 2.406%	242 ± 8.782%	48.41 ± 2.167%
**Training + EtOH**	122.3 ± 4.518%	196.4 ± 6.248%	66.39 ± 2.689%

NO: Nitrite level; LPO: lipid peroxidation levels; ACAP: antioxidant capacity against peroxyl

Supplementary Table S2: Amylase analysis and trolox-equivalent antioxidant capacity (TEAC) of saliva of mice exposed to ethanol in binge drinking model. Results are presented as mean ± standard error of mean.

	**Saliva**	
	**Amylase**	**TEAC**
**Control**	550.39 ± 29.9 U/dl	0.627 ± 0.0275 mM
**Training**	609 ± 11 U/dl	0.325 ± 0.020mM
**EtOH**	602.92 ± 16.81 U/dl	0.422 ± 0.026mM
**Training + EtOH**	563 ± 45 U/dl	0.446 ± 0.052mM

TEAC: trolox-equivalent antioxidant capacity
